# “Antimicrobial and antiproliferative activity of essential oil, aqueous and ethanolic extracts of *Ocimum micranthum* Willd leaves*”*

**DOI:** 10.1186/s12906-018-2122-z

**Published:** 2018-02-08

**Authors:** Isabel O. Caamal-Herrera, Leydi M. Carrillo-Cocom, Diana Y. Escalante-Réndiz, Diana Aráiz-Hernández, José A. Azamar-Barrios

**Affiliations:** 1 Departamento de Física Aplicada, Centro de Investigación y de Estudios Avanzados del IPN, Unidad Mérida, Km. 6 antigua carretera a Progreso, Apdo. Postal 73, Cordemex, C.P, 97310 Mérida, Yucatán México; 2 Cuerpo Académico de Biotecnología y Bioingeniería, Facultad de Ingeniería Química, Universidad Autónoma de Yucatán, Periférico Norte Kilómetro 33.5, Tablaje Catastral 13615, Chuburná de Hidalgo Inn, C.P, 97203 Mérida, Yucatán México; 3 Laboratorio de Cultivo Celular, Centro de Biotecnología FEMSA del Instituto Tecnológico y de Estudios Superiores de Monterrey, Campus Monterrey, Ave. Eugenio Garza-Sada 2501 Sur, Col. Tecnológico, C.P. 64849, Monterrey, N.L. México

**Keywords:** Antimicrobial, Proliferative, *Ocimum micranthum*, Leaves, Extracts, Essential oil

## Abstract

**Background:**

*Ocimum micranthum* Willd is a plant used in traditional medicine practiced in the region of the Yucatan peninsula. In particular, it is used for the treatment of cutaneous infections and wound healing, however there are currently no existing scientific studies that support these applications. The aim of the present study was to evaluate the antimicrobial and the in vitro proliferative activity (on healthy mammalian cell lines) of the essential oil and extracts (aqueous and ethanolic) of this plant.

**Methods:**

The minimal inhibitory concentration (MIC) of essential oil and aqueous and ethanolic extracts of *Ocimum micranthum* leaves against *Staphylococcus aureus, Bacillus subtilis, Pseudomonas aeruginosa* and *Candida albicans* was determined using the microdilution technique. The in vitro proliferative activity of human fibroblast (hFB) and Chinese hamster ovary (CHO-K1) cells treated with these extracts was evaluated using the MTT test. The hFB cell line was also evaluated using Trypan Blue assay.

**Results:**

*Candida albicans* was more susceptible to the ethanolic extract and the aqueous extract (MIC value of 5 μL/mL and 80 μL/mL respectively). In the case of *Staphylococcus aureus*, *Bacillus subtilis,* and *Pseudomonas aeruginosa*, the MIC of the aqueous and ethanolic extract was 125 μL/mL.

The aqueous extract showed a significant (*p* < 0.05) antiproliferative effect on hFB cells at a concentration of 4%, with cell proliferation percentage values of 73.56% and 20.59% by MTT method and Trypan Blue assay, respectively; the same effect was observed for the ethanolic extract at concentration from 0.06% to 0.25% using MTT method and at a concentration from 0.125% to 0.25% using Trypan Blue assay. In CHO-K1 cells an antiproliferative effect was observed at a concentration of 8% of aqueous extract and from 0.06% to 0.25% of ethanolic extract using the MTT method.

**Conclusion:**

These assays showed that low concentrations of essential oil and extracts of *Ocimum micranthum* leaves are sufficient to cause an antiproliferative effect on the hFB cell line but do not produce an antimicrobial effect against the microorganisms evaluated. More studies are necessary to improve understanding of the mechanism of action of the compounds implicated in the bioactivities shown by the crude extracts.

## Background

In Mexico, diverse plants have been utilized in traditional medicine in the treatment of several diseases, as well as in the treatment of cuts, cutaneous infections, wounds and burns [1, 2]. These presumptive curative properties may be attributable to secondary metabolites that the plants possess which are distributed in leaves, flowers, stems, seeds or roots [2]. Among the plants that have been reported to exert these biological activities are those that belong to the family *Lamiaceae* such as *Ocimum micranthum* Willd [3]. For other species of the genus *Ocimum* such as *sanctum linn*, *gratissimum linn*, *kilimandscharicum* wound healing properties of its crude extracts, and essential oil have been reported [4–6]; in the case of the crude extracts of the species *micranthum,* in vitro tests on healthy cell lines that support the therapeutic benefits of these extracts on wound healing and cutaneous infections have yet to be reported. Moreover, studies have not addressed the potential antimicrobial activity of these extracts against pathological microorganisms.

*Ocimum micranthum* Willd is an herbaceous native plant belonging to tropical and subtropical regions of America and the West Indies [7]. In Mexico, this plant is distributed in the states of Campeche, Chiapas, Colima, Jalisco, Oaxaca, Puebla, Queretaro, Quintana Roo, Sinaloa, Tabasco, Tamaulipas, Veracruz and Yucatan [8]. Previous studies have indicated that the essential oil of this species has activity against human pathogens, fungi, insects, and larvae in addition to its antioxidant, antiprotozoal, anti-inflammatory and contraceptive properties. It is believed that these properties may be related to the presence of diverse chemical compounds in the leaves of this plant [7, 9].

The chemical composition of the leaf oil of *Ocimum micranthum* Willd has been previously reported to comprise volatile compounds such as eugenol, β-elemene, γ-elemene β-caryophyllene, isoeugenol and methyl eugenol [10, 11]. The compounds eugenol and methyl eugenol have also been identified in aqueous and ethanolic extracts of this plant [11] and are phenolic derivatives commonly known for their use in cosmetic products (fragrances) and as flavoring agents in food products; both compounds have shown antiseptic, antibacterial and analgesic properties [12]. The effects of eugenol on mast cells and melanoma cells have been reported [13] and due to the broad field of application of this compound, it will be important to know its action on healthy human cells, such as skin cells (fibroblast and keratinocytes) [14].

Due to the chemical composition of the essential oil and extracts (ethanolic and aqueous) derived from the *micranthum* species, its therapeutic use in traditional medicine for the treatment of cutaneous infections and wounds, and since there are no scientific reports that support these bioactivities, the aim of the present study was to assess the essential oil and crude extracts (ethanolic and aqueous) of this plant for antimicrobial activity against some pathogenic microorganisms. In addition, the proliferative activity was assessed in vitro on a healthy human cell line (hFB) and the CHO-K1 cell line with the purpose of providing evidence (research-based) for its bioactivity and effects on a healthy cell line associated with the process of wound healing.

## Methods

### Plant material

The *Ocimum micranthum* Willd was taxonomically classified and identified by the biologist José L. Tapia of the Herbarium at Natural Resources Unit of the Center for Scientific Research of Yucatan, Merida, Yucatan, Mexico. A specimen was deposited in this same Herbarium with reference number 68785. Leaves of this plant were used to obtain the extracts evaluated in the present study. Leaves were collected during the winter season between December 2013 and February 2014 at 100 m around the point 21°9'10.91" North latitude and 89°5'4.58" West longitude in the town of Cansahcab, Yucatan, Mexico. Harvested leaves were washed and then dried in a convection oven at 50 °C for 16 h. Finally, the leaves were ground in a mill Ika (model A11).

### Preparation of essential oil and aqueous extract

The essential oil was obtained by hydro-distillation of ground leaves of *Ocimum micranthum* Willd, using a Clevenger trap [15, 16]. The fraction of essential oil was separated by density and then stored under refrigeration (4 °C) in glass vials sealed with Teflon tape, and covered with foil until its characterization.

The aqueous phase was collected in plastic containers, then filtered using a membrane of 0.22 μm and finally stored under refrigeration (4 °C). This phase was called the aqueous extract.

### Preparation of the ethanolic extract

The ethanolic extract was obtained by Soxhlet extraction of ground leaves of *Ocimum micranthum* Willd, using reagent-grade ethanol (JT Baker) as the solvent. The ethanol was recovered through Büchi rotary evaporator (model R-215) with a vacuum controller (V-850) coupled to a cooling unit. The extract was stored in glass bottles and then filtered through a filtration system comprising a stainless-steel base and a Millipore filter (0.22 μm). Finally, the ethanolic extract was labeled and refrigerated at 4 °C until its analysis.

### Microbial strains

Antimicrobial activity of the essential oil and extracts of *Ocimum micranthum* Willd leaves was evaluated through the determination of minimal inhibitory concentration (MIC) using the microdilution technique on a 96 well plate and by staining with a solution of iodonitrotetrazolium chloride (INT). The microorganisms used in this study consisted of two Gram-positive strains (*Staphylococcus aureus* ATCC® 25973TM and *Bacillus subtilis* ATCC® 465 TM); one Gram-negative strain (*Pseudomonas aeruginosa* ATCC ® 27,853 TM) and one yeast-fungus strain (*Candida albicans* ATCC® 14,053 TM).

### Growth kinetics

For the McFarland turbidimetric analysis [17], a wavelength of 590 nm was used. The absorbance values of this test were correlated with the absorbance values from the growth kinetics of each microorganism tested. This correlation was used to calculate the time taken by each microorganism to reach the exponential phase and the concentration of microorganism necessary to carry out the microdilution test. The value recorded in the present study was 0.50 on the McFarland scale which is equivalent to 1.5 × 10^8^ CFU/mL. In the growth kinetics, a pre-inoculum of each microorganism was incubated for 20–21 h at 35 °C in agitation. The culture medium used for this purpose were brain heart infusion (BHI) broth for *S. aureus* and *B. subtilis*, nutritive broth (*P. aeruginosa*) and sabouraud broth (*C. albicans*). The absorbance was measured every 2 h during a period of 16 h using a spectrophotometer model GENESYS 20 (®Thermo Scientific) at a wavelength of 590 nm.

### Minimal inhibitory concentration

In the MIC test, five concentrations of the fluid extracts (ethanolic, aqueous and essential oil) of *Ocimum micranthum* Willd leaves were analyzed. These concentrations were chosen through the evaluation of the results of osmolality and pH assays that were performed to avoid interferences in the tests with mammalian cells. The dilutions of the extracts were prepared with 5% dimethyl sulfoxide (DMSO) solution (D8418-500 mL ® Sigma Aldrich). This concentration of reagent was selected based on the results of a preliminary test where different concentrations of DMSO were evaluated to measure its toxicity on the microorganisms utilized in the present study and to eliminate the possibility of interference by the concentration of DMSO. Positive controls such as amikacin (4 mg/L) and nystatin (2 mg/mL), control of culture medium, color control of each extract concentration and positive control of growth of each microorganism were used. In the test, 100 μL of each microorganism suspension at a concentration of 1.5 × 10^8^ CFU/mL (0.5 of the McFarland scale) was inoculated in the 96 well microplates, and then 100 μL of each extract solution were added. The microplates were incubated at 35 °C for 20–21 h in the case of *S. aureus, B. subtilis*, and *P. aeruginosa*; in the case of *C. albicans*, the incubation time was 40–42 h. Once the incubation period had lapsed, 20 μL of a solution of iodonitrotetrazolium chloride 0.25 mg/mL (58030-1 g-F ®Sigma Aldrich) was added to the 96 well microplates, which were incubated at 35 °C for 1 h [18]. Subsequently, the MIC was determined visually, and of the wells that did not present a color change, an aliquot of 50 μL was taken to inoculate a Petri dish with a media corresponding to the evaluated microorganism. On the Petri dish, an extension technique using a digralsky spreader was carried out, after the Petri dishes were incubated at 35 °C for 24 h (*S. aureus, B. subtilis*, *and P. aeruginosa*) and 48 h (*C. albicans*). Finally, the microplates were read in a microplate reader (model Stat Fax 4200 (® Awareness Technology) at a wavelength of 492 nm.

The MIC was reported for each microorganism in every extract. The MIC was defined as the lowest concentration that led to growth inhibition, which was visually observed as no color change in the colorimetric test. The growth of some microorganisms in the Petri dishes indicated a bacteriostatic effect, while no growth of the microorganism indicated a bactericide effect of the extracts. Concerning *Candida albicans,* the terms that were applied were either a fungistatic or fungicide effect respectively.

### Measurement of pH and osmolality

Before performing the MTT test, the pH and osmolality of the culture media, which was supplemented with the extracts, were evaluated with the purpose of verifying that the values were in the optimum range and to avoid cytotoxic effects by osmotic shock or pH and, in this way, assess only the effect of the extracts on the cell lines. All osmolality measurements were performed with an osmometer (Advanced Instrument Inc. model 3320) using the freezing point depression method. The pH measurements were carried out with a VWR® SB90M5 pH meter.

### Cell lines and cell culture

Two cell lines were used in this study, healthy human breast-derived fibroblasts (hFB) and adherent Chinese hamster ovary cells (CHO-K1, Gibco, USA), this last cell line is a classic model of cytotoxic tests and proliferative assays due to its capacity of adaptation in adherent mode or suspension [19]. Both cell lines were routinely grown in DMEM F12 medium (Gibco, USA) supplemented with 10% fetal bovine serum (Gibco, USA) at 37 °C in a humidified atmosphere of 5% CO_2_.

### MTT assay

The effect of the essential oil and the aqueous and ethanolic extracts of *Ocimum micranthum* leaves on CHO-K1 and hFB cell lines was assessed using the tetrazolium colorimetric MTT assay [20]. The cells were seeded at a density 2 × 10^4^ cells/well in 96-well microtiter plates and incubated at 37 °C and 5% CO_2_ in a humidified environment for 24 h. Subsequently, 100 μL of six different concentrations of each extract was added to the wells, and the plates were incubated for 48 h. The final concentrations of the extracts in the wells were 0.0075%, 0.015%, 0.03%, 0.06%, 0.125% and 0.25% for essential oil; 0.25%, 0.5%, 1%, 2%, 4%,8% for aqueous extract and 0.0075%, 0.015%, 0.03%, 0.06%, 0.125% and 0.25% for ethanolic extract. After that, 10 μl of MTT solution (5 mg/ml in RPMI-1640 without phenol red, Sigma Aldrich®) was added to each well, and the plates were incubated at 37 °C for 2 h. Following the incubation period, 100 μL of MTT solvent (0.1 N HCl in anhydrous isopropanol) was added to the wells to solubilize the formazan crystals. Multiskan FC (®Thermo Scientific) microplate reader at 570 nm was used for the measurement of the absorbance. The measurement of the color control of all concentrations of each extract was carried out to ensure that no interference occurred in the measurement of each well. The percentage of relative cell proliferation was calculated based on a comparison with untreated cells (control) as [extract absorbance/control absorbance] × 100. Microscopic viewing of the cell cultures was performed before and after the assay using an inverted microscope Axio Vert 200 (Carl Zeiss) coupled to a video camera.

### Trypan blue exclusion assay

Trypan blue exclusion assay is a visual method used for the direct counting of viable cells. Therefore, it was chosen to evaluate the proliferative activity of the essential oil and extracts (aqueous and ethanolic) of *Ocimum micranthum* leaves on hFB cell line and for comparison with a colorimetric method such as MTT. The cells were seeded in a 24 well plate (cell density of 2 × 10^4^ cells/well) and incubated at 37 °C in an atmosphere of 5% CO_2_ and a humidified environment for 24 h. The next day, cells were treated with six different concentrations of each extract for 48 h. The morphological changes of treated and untreated cell line (control) were compared by monitoring, using an inverted microscope Axio Vert 200 (Carl Zeiss). After the morphological assessment, the cell viability was evaluated by Trypan blue dye exclusion assay. For this, the cells were rinsed with 1 mL of phosphate buffered saline (PBS 1X, Gibco) and trypsinized with 0.50 mL of 0.025% trypsin-EDTA (Gibco). Then, trypsin was neutralized by the addition of 0.50 mL of growth medium. Samples were taken and stained with 0.04% Trypan blue dye solution (Sigma Aldrich). Within two minutes, the cells were loaded in a Neubauer chamber, and the number of viable and non-viable cells in squares with a 1 mm^2^ area, was counted under a phase contrast microscope. The relative cell proliferation was determined as [no. of viable cells in the cells treated/ no. of viable cells in the cells no treated (control)] × 100.

### Statistical analysis

Results of MTT and Trypan Blue test were presented as the Mean ± Standard deviation (SD). The data were subjected to one-way analysis of variance (ANOVA) using STATGRAPHICS PLUS 5.1 statistical program. Duncan’s Method was used in the multiple comparisons in the cases where the ANOVA detected a significant difference (*p* < 0.05).

## Results

### Minimal inhibitory concentration

The results of the MIC test (Table 1) suggested that the fungi *Candida albicans* was the most susceptible microorganism to the ethanolic and aqueous extracts. The essential oil exerted the least antimicrobial effect which was indicated through displaying the highest value of MIC in all tested microorganisms. The ethanolic and aqueous extracts showed the same antimicrobial effect against *Staphylococcus aureus, Bacillus subtilis,* and *Pseudomonas aeruginosa,* microorganisms with different Gram, and therefore different cell wall chemistry. The positive controls, amikacin, and nystatin showed an antimicrobial effect at 4 mg/L and 2 mg/L respectively, because the wells of the plate, where the antibiotics were deposited with the different microorganisms, did not present a color change when the solution of iodonitrotetrazolium chloride was added during the MIC assay.

**Table 1:** Antimicrobial activity of essential oil and extracts of *Ocimum micranthum* Willd leaves

Plant	Extract	Minimal Inhibitory Concentration (μL/mL)			
		*Staphylococcus aureus*	*Bacillus subtilis*	*Pseudomonas aeruginosa*	*Candida albicans*
*Ocimum micranthum*	Ethanolic	125	125	125	5
	Aqueous	125	125	125	80
	Essential oil	500	250	500	125

The essential oil and the extracts (ethanolic and aqueous) of *Ocimum micranthum* leaves showed a bacteriostatic effect against *Staphylococcus aureus*, *Bacillus subtilis,* and *Pseudomonas aeruginosa*. The bacteriostatic effect against Gram-positive and Gram-negative bacteria, at the same concentration, suggests that the chemical composition of the extracts and its mechanism of action had a bacteriostatic effect that was independent of the chemistry of the cell wall of the microorganisms, which in other studies has been reported to be a factor.

In the case of *Candida albicans*, only the essential oil showed a fungicide effect, since its growth was not observed in the Petri dishes. These results suggest that the essential oil and the extracts of this plant may produce a fungicide or fungistatic effect at low concentrations in comparison with the levels that are necessary to cause a bacteriostatic effect. These last results are relevant since most of studies that have used the microdilution technique to calculate the MIC have not reported complementary tests necessary to verify the bactericide, bacteriostatic, fungicide or fungistatic effects of the extracts.

### MTT assay

The effect of the extracts on cell lines was determined through the calculation of the relative cell proliferation percentage in the MTT assay. The extract concentrations tested were chosen according to the results of the osmolality assay, where the optimum range for testing for cell proliferation is from 230 to 400 mOsm/kg. The parameter of the pH was also verified, and the optimum range, in this case, is from 6.60 to 7.80 [21].

**Fig. 1:**
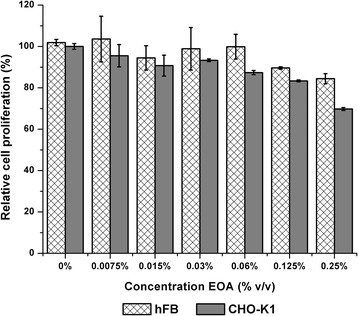
Effect of essential oil (EOA) on cell lines CHO-K1 and hFB by the MTT test. Error bars represent standard deviation

The results of the MTT test suggest that the essential oil produced a significant (*p* < 0.05) antiproliferative effect at concentrations of 0.06%, 0.125% and 0.25% on the CHO-K1 cell line with relative cell proliferation percentage values of 87.38%, 83.36% and 69.78% respectively (Fig. 1), while its effect on the fibroblast cell line did not significant (*p* > 0.05) in comparison with the control.

**Fig. 2:**
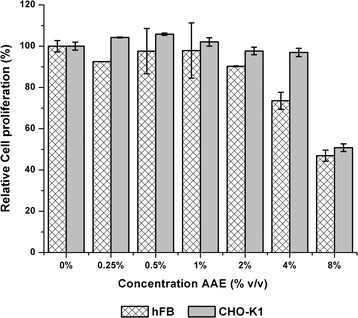
Effect of aqueous extract (AAE) on cell lines CHO-K1 and hFB by the MTT test. Error bars represent standard deviation

The aqueous extract of *Ocimum micranthum* Willd caused a decrease in the cell proliferation in both cell lines assessed (Fig. 2). At concentrations of 4% and 8%, the human fibroblast cell line displayed relative cell proliferation percentage values of 73.56% and 46.93% respectively. The same effect was observed on the CHO-K1 cell line but at a concentration of 8% with a relative cell proliferation percentage value of 50.82%. A slight proliferative effect was observed on the CHO-K1 cell line at concentrations of 0.25% and 0.50%.

**Fig. 3:**
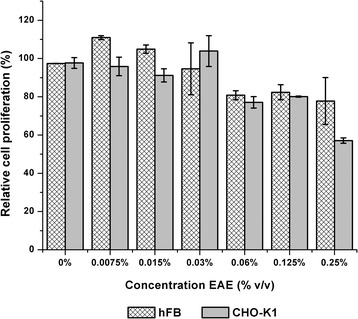
Effect of ethanolic extract (EAE) on cell lines CHO-K1 and hFB by the MTT test. Error bars represent standard deviation

The ethanolic extract from *Ocimum micranthum* decreased cell proliferation in both cell lines at low concentrations (Fig. 3). In the case of the human fibroblast cell line, this decrease was observed at concentrations of 0.06%, 0.125% and 0.25% with relative cell proliferation percentage values of 80.82%, 82.35%, and 77.80% respectively. The CHO-K1 cell line displayed this effect at the same concentrations of extract, but with values of 77.12%, 80.08%, and 57.12% respectively.

When the values of relative cell proliferation percentage were compared between the essential oil, aqueous and ethanolic extracts of *Ocimum micranthum* Willd, the aqueous extract demonstrated a significant (*p* < 0.05) antiproliferative effect on both cell lines at higher concentrations (4% on human fibroblast cell line and 8% on the CHO-K1 cell line) than the other extracts. While the ethanolic extract displayed the same effect at lower concentrations (from 0.06% *v*/v) in both cell lines.

### Trypan blue assay

**Fig. 4:**
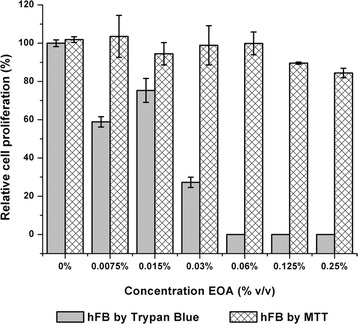
Comparative of relative cell proliferation percentage of hFB cell line in the essential oil (EOA) by MTT and Trypan blue test. Error bars represent standard deviation

The effect of the extracts on human fibroblasts was also evaluated using Trypan Blue assay. This technique permits the direct count of viable cells through the staining of the cells and microscopic observation. The results of this assay showed a notable difference concerning the MTT test. For example, the essential oil in the trypan blue test (Fig. 4) showed a relative cell proliferation less than 80% at all concentrations evaluated, in contrast to the results obtained in the MTT test, where the cell line presented values higher than 80% at all concentrations assayed. In the trypan blue test, all concentrations suggested a significant difference with a notable effect starting from a concentration of 0.0075%.

**Fig. 5:**
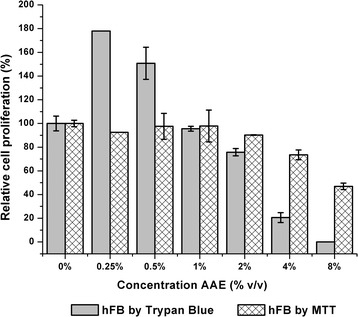
Comparative of relative cell proliferation percentage of hFB cell line in the aqueous extract (AAE) by MTT and Trypan blue test. Error bars represent standard deviation

The values of relative cell proliferation percentage of the aqueous extract (Fig. 5) showed a proliferative effect at concentrations of 0.25% and 0.50% *v*/v in the Trypan blue test, although the MTT test did not show a significant difference compared to the control. However, in both tests there was a tendency of the relative cell proliferation percentage to decrease, starting at a concentration of 2% *v*/v of the extract. The decrease in the values of the relative cell proliferation percentage in the trypan blue assay was higher than in the MTT test.

**Fig. 6:**
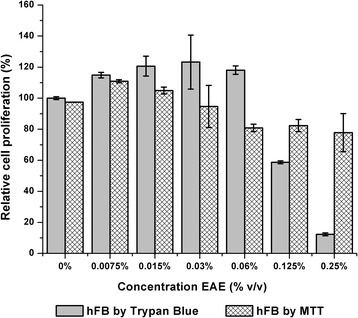
Comparative of relative cell proliferation percentage of hFB cell line in the ethanolic extract (EAE) by MTT and Trypan blue test. Error bars represent standard deviation

The values of relative cell proliferation percentage of the ethanolic extract at low concentrations (Fig. 6) showed a similar behavior in both tests (Trypan blue and MTT). A slight significant (*p* < 0.05) proliferative effect concerning the control was observed in the Trypan Blue and MTT tests at concentrations of 0.0075% and 0.015% *v*/v. However, in the Trypan Blue assay the proliferative effect remained until a concentration of 0.06% v/v, while in MTT test a decrease of cell proliferation was observed from this concentration. Finally, when extract concentrations of 0.125% and 0.25% v/v were tested using the trypan blue assay, relative cell proliferation percentage values of 58.71% and 12.26% respectively were obtained, while in the MTT test the values of relative cell proliferation percentage were 82.35% and 77.80% respectively.

## Discussion

Some studies have reported the antimicrobial activity of essential oil from diverse species of the *Ocimum* genus, such as *micranthum* and *basilicum* using the diffusion disc method [22]. In these studies, the essential oil from *micranthum* species showed higher antimicrobial activity against *C. albicans* (0.069 mg/L) and *P. aeruginosa* (0.173 mg/L) than essential oil from the *basilicum* species, however, *basilicum* species showed higher antimicrobial activity against *S. aureus* (0.057 mg/L) than *micranthum* species (0.104 mg/L). Other authors [23] have reported antimicrobial activity of the ethanolic extract from *Ocimum basilicum* using the micro-well dilution method, where this extract had a MIC of 250 μg/mL against *S. aureus*, however, activity was not demonstrable against *Candida albicans*. These authors also used the diffusion disc method, where the ethanolic extract showed activity against *S. aureus* (8 mm) and *Bacillus subtilis* at a concentration of 300 μg/disc, but the extract did not show activity against *P. aeruginosa* and *C. albicans.*

Other studies [24] have observed the antimicrobial activity of the ethanolic extract from *Ocimum gratissimum* leaves against *P. aeruginosa, S. aureus* and antifungal activity against *C. albicans*; these activities were increased when the concentration of the extract also increased. Other authors [25] have reported antimicrobial activity against *S. aureus,* and *P. aeruginosa* exerted by the ethanolic extract from *Ocimum sanctum* using the diffusion disc method. There is a study that reported higher inhibition activity of the ethanolic extract from *Ocimum basilicum* against *S. aureus* and *E. coli* at 200 mg/L using the hole-plate diffusion method [26].

Other authors carried out a study of the antifungal activity of essential oils derived from diverse species of the *Ocimum* genus (*americanum*, *basilicum* variety *purpurascens*, *basilicum* variety *minimum*, *micranthum*, *selloi)* against diverse species of the *Candida* genus using broth microdilution method, in accordance with the Clinical and Laboratory Standards Institute-CLSI [27]. In this study, it was observed that essential oil from *americanum*, *basilicum* variety *purpurascens* and *basilicum* variety *minimum* species did not display inhibitory activity against *C. albicans* (ATCC 3719). The essential oil from *americanum* and *basilicum* variety *purpurascens* had high MIC values (5000 μg/mL) against *C. albicans* (ATCC 11006), while *basilicum* variety *minimum* did not show inhibitory activity. The essential oil from *selloi* species showed inhibitory activity against *C. albicans* (ATCC 3719) and *C. albicans* (ATCC 11006) at a concentration of 1250 μg/mL. The essential oil from the *micranthum* species displayed inhibitory activity against *C. albicans* 3719 and *C. albicans* (ATCC 11006) at concentrations of 1250 μg/mL and 625 μg/mL respectively.

The variation of the antimicrobial activity of several species of *Ocimum* genus as well as the same *micranthum* species may be attributed to the biochemical properties of the plants that have been influenced by several factors such as the geographical origin, soil, environmental conditions, crop conditions, and seasonal variations. This may also be linked to the difference in the chemical composition, especially the presence of eugenol, since aromatic alcohols are mainly responsible for the antimicrobial activity of essential oils [27, 28]. Some authors have mentioned that the antimicrobial action of essential oil is due to the lipophilic character of its hydrocarbon skeleton and the hydrophilic character of its functional groups; the chemical group with higher antimicrobial activity is phenol, followed by aldehydes, ketones, alcohols, ethers, and hydrocarbons [27]. A higher antimicrobial activity has been reported for phenolic compounds such as thymol, carvacrol, and eugenol, which is associated with the acidic nature of the hydroxyl group, forming a hydrogen bond with an enzyme active center [29]. Concerning the volatile compounds in the essential oil from the *micranthum* species, some authors [8] used the GC-MS analysis to identify majority compounds such as β-caryophyllene (27%), methyl eugenol (14%), eugenol (12%) and in lower percentages, spathulenol (3%) and caryophyllene oxide (3%). Also, the authors identified compounds with antimicrobial activity in the ethanolic extract such as eugenol (18%), β-caryophyllene (6%), benzoic acid (3%), methyl eugenol (2%), dodecanoic acid (2%) and spathulenol (1%). Finally, in the aqueous extract eugenol (59%), 2,2-dimethyl-4-(methylethyl)-2H–imidazole, (4%), phenethyl alcohol (2%), methyl eugenol (2%) and catechol (1%) were identified, compounds that have shown antimicrobial activity [30, 31]. In the present study, the ethanolic extract showed a bacteriostatic effect and a fungistatic effect at lower concentrations than the aqueous extract, despite having the lowest content of eugenol, thus it is possible that the antimicrobial properties of the ethanolic extract may be attributed to a synergic effect between its compounds.

The behavior of the ethanolic extract in the MTT assay can be predicted from the MIC test where a fungistatic effect on *Candida albicans* at a low concentration (5 μL/mL) was observed; this fungus is a microorganism of eukaryote origin as the cell lines that were used in MTT assay. This behavior may be associated with the chemical nature of the extract, specifically a possible synergic effect of its compounds, because this extract has a lower percentage of eugenol, a phenolic compound with high antimicrobial activity and antiproliferative activity on cancer cells. Eugenol has been shown to be a molecule capable of exerting an antiproliferative effect on diverse cancer cells [32], however, it does not seem to be the determinant compound in the antiproliferative behavior of *Ocimum micranthum* extracts, since the aqueous extract exerted this effect at higher concentrations, despite containing a higher content of eugenol than the essential oil and the ethanolic extract [11].

All results obtained in the Trypan blue assay were coherent with the visual observations made under the microscope, while this did not occur with the results of the MTT test. Also, through visual inspection, the cytotoxic effect of the extracts on hFB cells was observed.

In general, in the MTT assay, the antiproliferative effect on the human fibroblast cell line was underestimated when high concentrations of the extracts from *Ocimum micranthum* Willd were tested. The results also suggest that the measurement of the antiproliferative or proliferative effects of the phytochemicals contained in the extracts may vary between a colorimetric method (MTT assay) and a method that involves the direct counting of viable cells such as the Trypan blue assay. This difference is probably associated with the interaction of diverse chemical components in the extracts (such as the phenolic compounds) with the MTT reagent [33]; it is possible that these types of compounds may interfere with critical formazan formation in the MTT method.

In a previous study [34], it was observed that natural compounds with intrinsic reductive potential such as some flavonoids, phytoestrogens and ascorbic acid might lead to false positives in the MTT assay, due to a mechanism of non-enzymatic reduction of the MTT to formazan. Other authors have reported that changes in the metabolism of the cells may induce an increase in the reduction of the MTT to formazan, which has been observed as an increase in the coloration of the reaction, hence in the values of absorbance [35].

In the comparison of the three extracts, it was observed that the underestimation of the antiproliferative effect was more notable in the essential oil, which may be related to a synergic effect of some volatile compounds with antioxidant characteristics present in the extract such as eugenol, methyl eugenol and other compounds including isoborneol, eucalyptol, spathulenol, a profile of volatile compounds that have been previously identified [11]. The isolation of the active compounds from these extracts, as well as in vivo studies are necessary, and that can improve understanding of the mechanisms underlying these bioactivities.

## Conclusion

These assays showed that low concentrations of essential oil and extracts of *Ocimum micranthum* leaves are sufficient to cause an antiproliferative effect on the hFB cell line but do not produce an antimicrobial effect against the microorganisms evaluated in this study, whereby neither of the extracts demonstrated both bioactivities at the same time. However, the ethanolic extract showed potential as a fungistatic agent at low concentrations. These results have also indicated the importance of conducting studies on the effects of natural extracts, which are used in traditional herbal medicine, on proliferation or cytotoxicity using in vitro tests and healthy cell lines.

More studies are necessary to improve understanding of the mechanism of action of the compounds implicated in the bioactivities shown by the crude extracts.

## Abbreviations

ATCC: American Type Culture Collection
*B. subtilis*: *Bacillus subtilis*
*C. albicans*: *Candida albicans*
CHO-K1: Chinese hamster ovary
DMSO: Dimethyl sulfoxide
*E. coli*: *Escherichia coli*
GC-MS: Gas chromatography–mass spectrometry
hFB: Human fibroblast
INT: P-iodonitrotetrazolium
MIC: Minimal inhibitory concentration
*P. aeruginosa*: *Pseudomonas aeruginosa*
*S. aureus*: *Staphylococcus aureus*
SD: Standard Deviation

## References

[1] González-Elizondo M, López-Enríquez L, González-Elizondo S, Tena-Flores J. Plantas Medicinales del Estado de Durango y Zonas Aledañas. Centro Interdisciplinario de Investigación para el Desarrollo Integral Regional (CIIDIR) Unidad Durango. Instituto Politécnico Nacional. México, D.F. 2004.p. 12–13.

[2] Sánchez-Medina A, García-Sosa K, May-Pat F, Peña-Rodríguez LM (2001). Evaluation of biological activity of crude extracts from plants used in Yucatecan traditional medicine. Part1. Antioxidant, antimicrobial and ß-glucosidase inhibition activities. Phytomedicine.

[3] De Pinho JPM, Silva ASB, Pinheiro BG, Sombra I, De Carvalho-Bayma J, Lauhlou S (2012). Antinociceptive and antispasmodic effects of the essential oil of *Ocimum micranthum*: potential anti-inflammatory properties. Planta Med.

[4] Shetty S, Udupa S, Udupa L. Evaluation of antioxidant and wound healing effects of alcoholic and aqueous extract of *Ocimum sanctum linn* in rats. Evid Based Complement Alternat Med (*e* Cam) 2008; 5(1): 95–101. 10.1093/ecam/nem004 PMC2249741 18317555

[5] Orafidiya LO, Agbani EO, Abereoje OA, Awe T, Abudu A, Fakoya FA (2003). An investigation into the wound-healing properties of essential oil of *Ocimum gratissimum linn*. J Wound Care.

[6] Paschapur M, Patil MB, Kumar R, Evaluation PSR (2009). Of aqueous extract of leaves of *Ocimum kilimandscharicum* on wound healing activity in albino wistar. Int J PharmTech Res.

[7] Lino CS, Gomes PB, Lucetti DL, Diógenes JPL, Sousa FCF, Silva MGV (2005). Evaluation of Antinociceptive and antiinflammatory activities of the essential oil (EO) of *Ocimum micranthum* Willd from northeastern Brazil. Phytother Res.

[8] Villaseñor JL, Espinosa FJ (1998). Catálogo de malezas de México.

[9] Jaramillo BE, Duarte E, Delgado W (2014). Bioactividad del aceite esencial de *Ocimum micranthum* Willd recolectado en el departamento de Bolívar. Colombia Rev Cubana Plant Med.

[10] Silva MGV, Craveiro AA, Matos FJA, Machado MIL, Alencar JW, Aurelio FKF (1998). Essential oils from leaves and inflorescences of *Ocimum micranthum* Willd from northeastern Brazil. J Essent Oil Res.

[11] Caamal-Herrera IO, Muñoz-Rodríguez D, Madera-Santana T, Azamar-Barrios JA (2016). Identification of volatile compounds in essential oil and extracts of *Ocimum micranthum* Willd leaves using GC/MS. Int J Appl Res Nat Prod.

[12] Ghosh R, Nadiminty N, Fitzpatrick JE, Alworth WL, Slaga TJ, Kumar AP (2005). Eugenol causes melanoma growth suppression through inhibition of E2F1 transcriptional activity. J Biol Chem.

[13] Park BS, Song YS, Yee SB, Lee BG, Seo SY, Park YC (2005). Phospho-ser 15-p53 translocates into mitochondria and interacts with Bcl-2 and Bcl-xL in eugenol-induced apoptosis. Apoptosis.

[14] Kalmes M, Blömeke B (2012). Impact of Eugenol and Isoeugenol on AhR translocation, target gene expression and proliferation in human HaCaT keratinocytes. J Toxicol Environ Health A.

[15] Charles DJ, Simon JE, Wood KV (1990). Oil constituents of *Ocimum micranthum* Willd. J Agric Food Chem.

[16] Rodríguez-Álvarez M, Alcaraz-Meléndez L, Real-Cosío SM. Procedimientos para la extracción de aceites esenciales en plantas aromáticas. Edit. Centro de Investigaciones Biológicas del Noroeste, S.C. La Paz, Baja California Sur, México. 2012. p.24–29.

[17] McFarland J (1907). The Nephelometer: an instrument for estimating the number of bacteria in suspensions used for calculating the opsonic index and for vaccines. J Am Med Assoc.

[18] Eloff JN (1998). A sensitive and quick microplate method to determine the minimal inhibitory concentration of plant extracts for bacteria. Planta Med.

[19] Sestili P, Cantoni O, Cattabeni F, Murray D (1995). Evidence for separate mechanisms of cytotoxicity in mammalian cells treated with hydrogen peroxide in the absence or presence of L-histidine. Biochim Biophys Act.

[20] Mosmann T (1983). Rapid colorimetric assay for cellular growth and survival: application to proliferation and cytotoxicity assays. J Immunol Methods.

[21] Goswami M, Chaitra TR, Chaudhary S, Manuja N, Sinha A (2011). Strategies for periodontal ligament cell viability: an overview. J Conserv Dent.

[22] Saccheti G, Medici A, Maletti S, Radice M, Muzzoli MV (2004). Composition and functional properties of the essential oil of Amazonian basil, *Ocimum micranthum* Willd Labiatae in comparison with commercial essential oils. J Agric Food Chem.

[23] Adigüzel A, Güllüce M, Sengüil M, Ögütcü H, Sahin T, Karaman I (2005). Antimicrobial effects of *Ocimum basilicum* (Labiatae) extract. Turk J Biol.

[24] Oboh G (2010). Antioxidant and Antimicrobial properties of ethanolic extracts of Ocimum gratissimum leaves. J Pharmacol Toxicol.

[25] Joshi B, Sah GP, Basnet BB, Bhat MR, Sharma D (2011). Phytochemical extraction and antimicrobial properties of different medicinal plants: *Ocimum sanctum* (Tulsi), *Eugenia caryophyllata* (clove), *Achyranthes bidentata* (Datiwan) and *Azadirachta indica* (Neem). J Microbiol Antimicrob.

[26] Khalil A (2013). Antimicrobial activity of Ethanolic extracts of *Ocimum basilicum* leaf from Saudi Arabia. Biotechnology.

[27] Vieira P, M de Morais S, Bezerra HQF, Travassos-Ferreira PA, Oliveira IR, Silva MGV (2014). Chemical composition and antifungal activity of essential oils from Ocimum species. Ind Crop Prod.

[28] Fontenelle RO, Morais SM, Brito EH, Brilhante RS, Cordeiro RA, Lima YC (2011). Alkylphenol activity against *Candida* spp. and *Microsporum canis*: a focus on the antifungal activity of thymol, eugenol and O-methyl derivatives. Molecules.

[29] Kalemba D, Kunicka A (2003). Antibacterial and fungi properties of essential oils. Curr Med Chem.

[30] Gutierrez J, Barry-Ryan C, Bourke P (2009). Antimicrobial activity of plant essential oils using food model media: efficacy, synergistic potential and interactions with food components. Food Microbiol.

[31] Rahman A, Shanta ZS, Rashid MA, Parvid T, Afrin S, Khatun MK, et al. *vitro* antibacterial properties of essential oil and organic extracts of *Premna integrifolia* Linn. Arab J Chem. 2011; 10.1016/j.arabjc.2011.06.003.

[32] Jaganathan SK, Supriyanto E (2012). Antiproliferative and molecular mechanism of Eugenol-induced apoptosis in cancer cells. Molecules.

[33] Wang P, Henning SM, Heber D (2010). Limitations of MTT and MTS-based assays for measurement of Antiproliferative activity of green tea polyphenols. PLoS One.

[34] Bruggisser R, von Daeniken K, Jundt G, Schaffner W, Tullberg-Reinert H (2002). Interference of plant extracts, phytoestrogens, and antioxidants with MTT tetrazolium assay. Planta Med.

[35] van Tonder A, Joubert AM, Cromarty AD (2015). Limitations of the 3-(4,5-dimethylthiazol-2-yl)- 2,5-diphenyl-2H-tetrazolium bromide (MTT) assay when compared to three commonly used cell enumeration assays. BMC Res Notes.

